# Monotonicity in graph theoretic summaries of fMRI data acquired during human learning

**DOI:** 10.3389/fnhum.2025.1595331

**Published:** 2025-09-22

**Authors:** Dhruval Bhatt, John Kopchick, Clifford Abel, Patricia Thomas, Usha Rajan, Dalal Khatib, Caroline Zajac-Benitez, Luay Haddad, Alireza Amirsadri, Jeffrey A. Stanley, Vaibhav A. Diwadkar

**Affiliations:** ^1^Department of Psychiatry and Behavioral Neurosciences, Wayne State University, Detroit, MI, United States; ^2^Surgical Neurology Branch, National Institutes of Health, Bethesda, MD, United States; ^3^Translational Neuroscience Program, Detroit, MI, United States

**Keywords:** monotonicity, graph theory, learning, behavior, fMRI

## Abstract

**Introduction:**

Behavioral performance during associative learning typically improves monotonically; performance on each successive iteration of the task is no worse (and typically better) than on the previous one. It is unclear whether connectomic measures of brain function (from fMRI data acquired during learning) also increase monotonically. We used a well-established associative learning paradigm to test for the possible co-observance of monotonicity in behavior and connectomics.

**Methods:**

fMRI data were summarized using two distinct connectomic (i.e., graph theoretic) measures: (a) Betweenness Centrality (of nodes) and (b) Average Shortest Path Length (i.e., a measure of network efficiency) across the graph. To broaden our study’s breath, in addition to healthy controls (*n* = 39), we extended the analyses to data collected in schizophrenia patients (*n* = 49). Past studies show that although patients show deficits in learning (lower learning capacity), behavior does typically display monotonicity.

**Results:**

We observed robust evidence for monotonic changes in behavior at the group level, and in most participants regardless of group. Evidence for monotonic changes in graph theoretic summaries of the co-acquired fMRI data was less widespread and was in general, more evident in group level summaries (regardless of group).

**Discussion:**

This modest co-observance of monotonicity in behavior and fMRI-based connectomics re-emphasizes what has long been suspected: the relationship between overt measures of behavioral competence and the co-acquired imaging signals is complex. This may be because psychological events (whether in the healthy brain, or in clinical populations like schizophrenia) emerge not from local activity in circumscribed brain regions, but rather from widely distributed activity across the brain. While well-defined mathematical concepts like monotonicity can anchor attempts to co-observe properties of change in overt behavior, and underlying brain signals, we suggest that the search for such relationships will remain a challenge.

## 1 Introduction

In mathematics, monotonic functions are those that preserve the mapping (forward or inverse) between ordered sets (Merkle, 2014). More generally, monotonicity reveals itself to be a feature of many psychological and biological settings (Grice et al., 2023). For instance, monotonicity is a frequently observed property of *behavioral* functions, wherein a set of measurable behavioral responses are often systematically mapped to some change in the stimuli or the task that the behavior results from Shepard (1987). In psychophysics, the *perceived* increase in the intensity of a stimulus is a monotonic function of changes in actual stimulus intensity (Luce, 2002). Monotonicity is also observed “up” the cognitive hierarchy. Studies of mental rotation (of visual objects or scenes) show that the time needed to decide if two objects or scenes are identical is a monotonic function of the three dimensional rotation required to align the two objects or scenes in space (Shepard and Cooper, 1986; Diwadkar and McNamara, 1997; McNamara et al., 2006). Finally, and pertinent to this investigation, experimental *learning* tasks evoke at least weakly monotonic behavioral performance; typically, behavioral proficiency on each task iteration is no worse, or better than on the previous one (Gallistel et al., 2004; Diwadkar et al., 2008). Such monotonicity is widely observed in frontal-hippocampal based human learning and memory tasks (e.g., where participants must learn associations between two arbitrarily paired memoranda such as objects and locations) (Büchel et al., 1999; Mattfeld and Stark, 2015; Stanley et al., 2017). Interestingly weakly monotonic learning is preserved even in pathologies like schizophrenia (SCZ), where learning capacity is reduced (Diwadkar et al., 2008; Brambilla et al., 2011; Meram et al., 2023). Given such evidence, it is plausible that changes in fMRI signals collected during learning might also show evidence of monotonicity, where fMRI metrics increase or decrease over the course of learning. However, to our knowledge, few investigations of this question exist. This lacuna is significant; any resultant observations may not directly shed light on the vexing question of brain – behavior relationships (Westlin et al., 2023). However, monotonicity can be a mathematical anchor using which one can investigate changes in fMRI data co-acquired in specific task contexts (Solo et al., 2018). Accordingly, our investigation specifically addressed two questions: (1) is monotonicity of fMRI data collected during learning (Wadehra et al., 2013; Woodcock et al., 2015; Woodcock et al., 2016) observed in *summary* measures (based on graph theory) of such data? (2) Is such monotonicity in the fMRI data observed in both (or neither) healthy participants and in a clinical group like schizophrenia, in whom learning proficiency is impaired but behavioral monotonicity is generally preserved?

**Figure 1:**
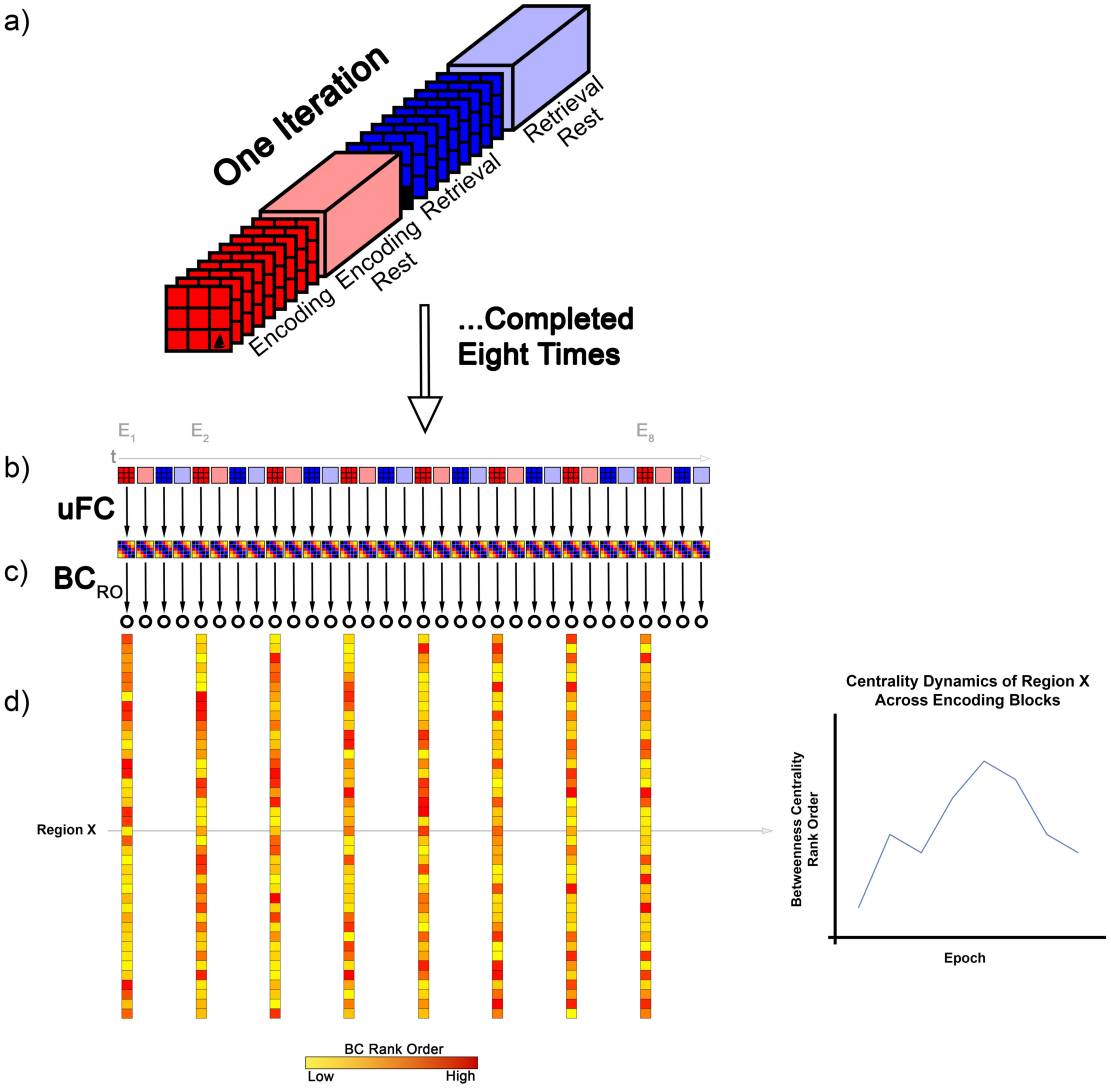
The figure provides a schematic depiction of the analytical approach to our study. **(a)** The four conditions (Encoding, Post- Encoding Rest, Retrieval, and Post-Retrieval Rest) within each iteration of the associative learning task are depicted. As noted, (Methods), there were eight repetitions. Briefly, during Encoding, objects were presented in their associated location for naming (e.g., “tree”). During Retrieval, locations were cued in random order and participants were required to name the associated object. Rest intervals were interspersed between. **(b)** For each condition and in each iteration, a uFC matrix was computed (schematically depicted at 5 × 5; actual size 246 × 246, 30,135 unique functional connections) based on extracted time series. In each participant, eight such matrices were computed per condition. **(c)** Treating the matrices as undirected weighted graphs, we next computed the BC for each node (represented by the open circle) in each graph. **(d)** Next, nodes were rank ordered by BC (BC_RO_). In the schematic, we depict the BC_RO_, for each node in each of the eight Encoding conditions (E_1_ to E_8_). As seen, Region X showed a non-monotonic change in BC_RO_ across the task (graph at right).

fMRI data were co-acquired as participants performed an established associative learning task (see Figure 1). The task required participants to learn object-location associations over eight successive task epochs (Stanley et al., 2017). As noted, the observed learning functions are typically monotonic, not just at the group level, but also generally at the individual participant level (Büchel et al., 1999; Samona et al., 2024), where performance on each successive task epoch is typically *no worse* than on the preceding epoch. In our investigation, we used multiple imaging-related outcome measures, with a principle focus on *graph-theoretic summaries* of the fMRI data.

### 1.1 The choice of graph theory

Graph theory is a leading tool for summarizing the characteristics of functional networks at multiple scales (Sporns, 2014). In the context of fMRI data, graph theoretical measures are typically applied to summarize network properties after the fMRI time series data are processed through statistical measures like functional connectivity (that capture consistencies in the behavior of brain regions) (Friston, 2011; Silverstein et al., 2016). Graph theory lucidly characterizes large scale connectomic profiles into local or global topological measures (Freeman, 1977; Meram et al., 2023; Huang et al., 2024). Notably, the topological characteristics of brain networks show some relationship to performance and adaptation during tasks such as motor learning, where for example, the modular organization of networks adapts as learning proficiency increases (Bassett et al., 2011). Our investigation included two complementary graph-theoretic measures: (a) Betweenness Centrality (BC) which quantifies *a node’s role* as a bridge along the shortest path between any two other nodes (Freeman, 1977), and (b) Average Shortest Path Length (ASPL) which summarizes the degree of integration *across the network* (Betzel et al., 2016). BC is highly sensitive to any node’s relative importance within a network (Rubinov and Sporns, 2010) because it quantifies a nodes’ integrative value (Kivimaki et al., 2016; Meram et al., 2023). By comparison, ASPL offers a *connectome-level* measure of integration indicating how closely a network is connected (Mao, 2013).

Learning proficiency was assessed over eight successive epochs with each epoch divided into four separate task conditions (see Methods) (Hasan et al., 2023; Martin et al., 2024). After parcellating the brain using a multi-modal 246-region cerebral parcellation scheme (Fan et al., 2016; Zhi et al., 2018), separate undirected graphs were derived for each task condition and epoch (eight graphs per condition, with one at each time point, i.e., epoch). Each such graph consisted of 246 nodes (one for each region) and 30,135 unthresholded edges (where edge weights were the zero-lag functional connectivity values between those regions) (Silverstein et al., 2016). For each region/node and participant, we estimated BC in each condition and epoch. We could form a resultant function for each node, where each such function represented the change in BC over the eight epochs of the task for the given condition. These functions were investigated for evidence of monotonicity. In complementary analyses, ASPL was estimated for each epoch (following which we again searched for evidence of monotonicity in the formed functions). These analyses were conducted at the level of individual participants. Subsequent analyses were also extended to the group level (using group-averaged graphs). Analyses were conducted separately for HC and SCZ on the presumption that monotonicity may be *less likely* to be observed in SCZ. Finally, in supplementary analyses, we searched for evidence of monotonicity in regional changes in fMRI signal amplitude.

**Figure 2:**
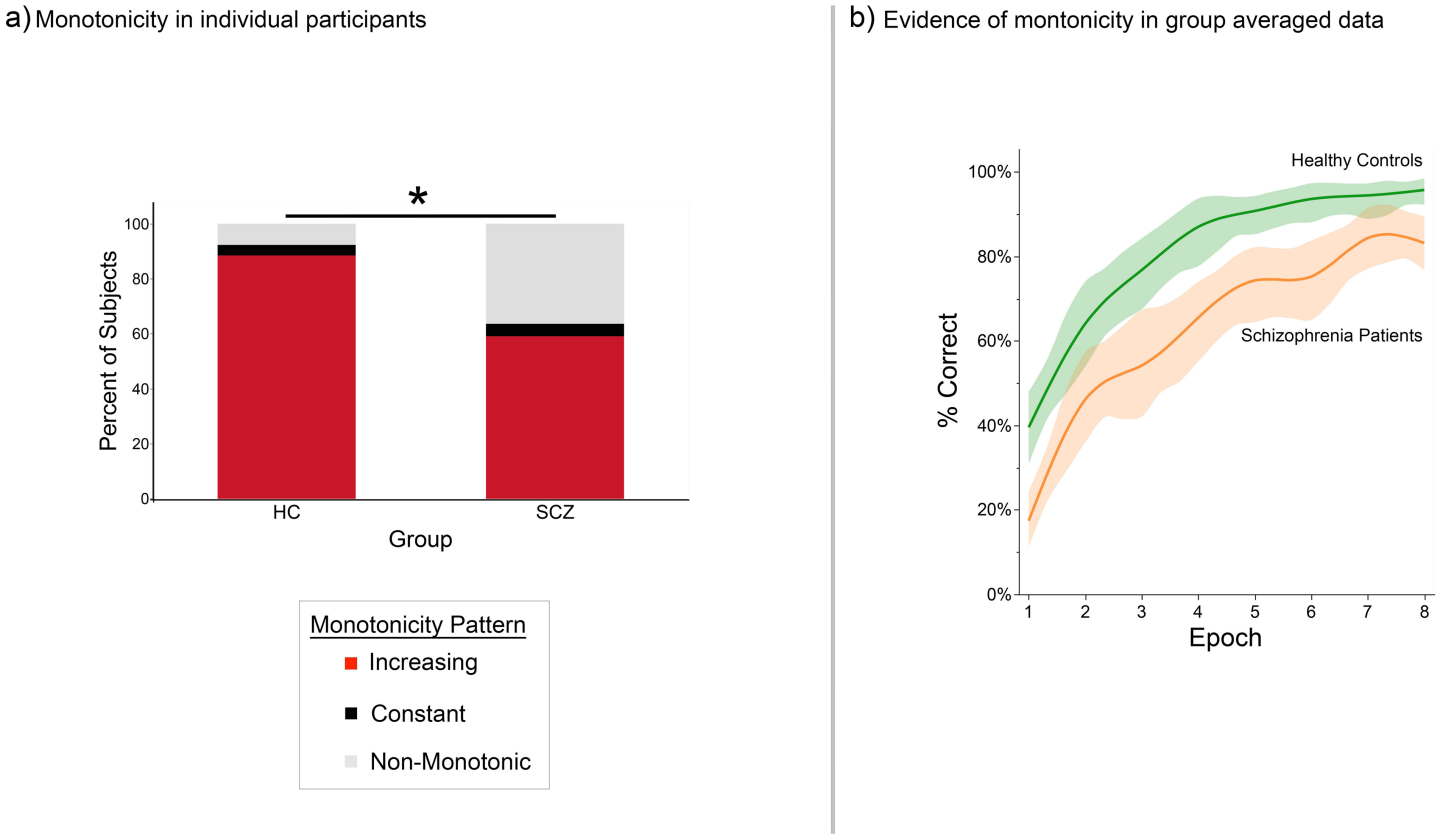
Monotonic changes in behavioral proficiency (proportion correct recall) across the task in **(a)** individual participants and **(b)** group performance functions. **(a)** The stacked bar graphs depict the percentage of participants in each group who displayed either monotonic (red sub-bar) or non-monotonic changes in behavioral proficiency. Most of the healthy controls and a majority of patients exhibited monotonic increases in behavioral proficiency (though the percentage of control participants who displayed monotonicity was higher, Fisher’s Exact Test, *p* < 0.05). **(b)** Group averaged performance functions accentuated these trends. Here, average percent correct performance (vertical axis) is depicted in each of the eight iterations (horizontal axis). The data are smoothed using LOESS (*λ* = 0.0000126, with 95% confidence intervals shown).

**Figure 3:**
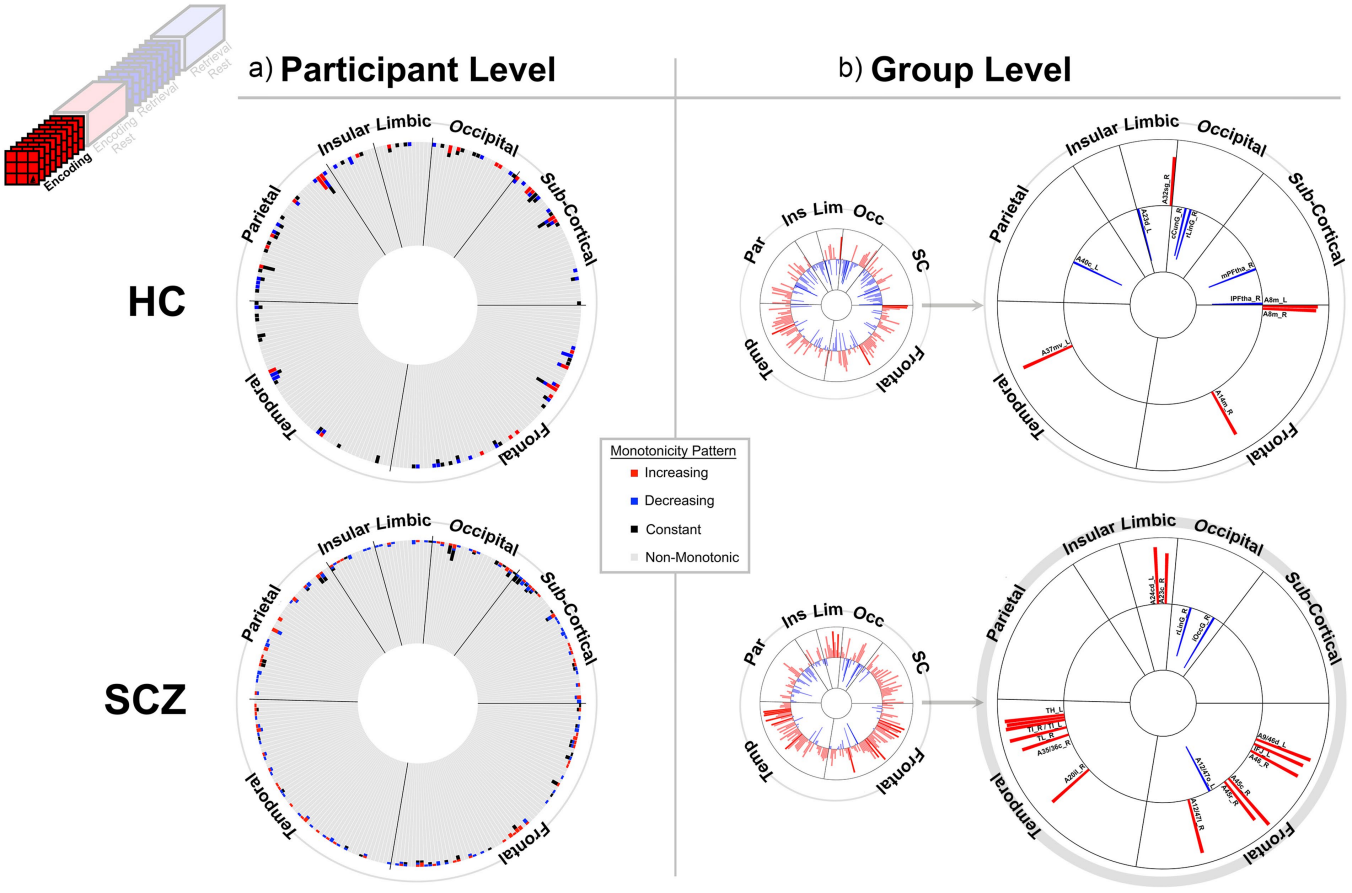
Monotonicity of BC_RO_ during Encoding at the **(a)** participant and **(b)** group level. **(a)** The 246 brain regions (nodes) are arranged in a circular arrangement, grouped by lobe (regional names are withheld to reduce clutter). A stacked frequency bar is attached to each region, where each bar represents the frequency of subjects showing patterns of weak monotonicity or non-monotonicity for any node (see color bar in the center of the figure). In both groups non-monotonicity of BC_RO_ was the norm (HC: 97.7%, SCZ: 97.8%). **(b)** Group level evidence (see Methods). Regions are arranged as in **(a)**. Each bar represents the Spearman’s ρ for that region and group (red and blue bars, respectively, represent positive and negative monotonicity). The small circles represent data from all 246 regions. These are statistically filtered (*p* < 0.05) and presented in the larger circles to the right (region names are added). We observed some monotonic effects at the group level, with more regions showing positive monotonicity in SCZ (bottom). More descriptive region labels follow based on Fan et al. (2016). HC: A32sg_R, Cingulate Gyrus sub genual area 32; A23d_L, Cingulate Gyrus dorsal area 23; cCunG_R; MedioVentral Occipital caudal cuneus gyrus; rLinG_R; MedioVentral Occipital Cortex rostral lingual gyrus; mPFtha_R, Thalamus medial pre-frontal thalamus; lPFtha_R, Thalamus lateral pre-frontal thalamus; A8m_L, Superior Frontal Gyrus medial area 8; A8m_R, Superior Frontal Gyrus medial area 8; A14m_R, Orbital Gyrus medial area 14; A37mv_L, Fusiform Gyrus medioventral area37; A40c_L, Inferior Parietal Lobule caudal area 40(PFm). SCZ: A24cd_L, Cingulate Gyrus caudodorsal area 24; A23c_R, Cingulate Gyrus caudal area 23; rLinG_R, MedioVentral Occipital Cortex rostral lingual gyrus; iOccG_R, lateral Occipital Cortex inferior occipital gyrus; A9/46d_L, Middle Frontal Gyrus dorsal area 9/46; IFJ_L, Middle Frontal Gyrus inferior frontal junction; A45c_R, Inferior Frontal Gyrus caudal area 45; A12/47o_L, Orbital Gyrus orbital area 12/47; A12/47l_R, Orbital Gyrus lateral area 12/47; A20il_R, Inferior Temporal Gyrus intermediate lateral area 20; TH_L, Parahippocampal Gyrus area TH (medial PPHC); TI_R, Parahippocampal Gyrus area TI (temporal agranular insular cortex); TI_L, Parahippocampal Gyrus area TI (temporal agranular insular cortex); A35/36c_L, Parahippocampal Gyrus caudal area 35/36; TL_R, area TL (lateral PPHC, posterior parahippocampal gyrus); A35/36c_R, Parahippocampal Gyrus caudal area 35/36.

**Figure 4:**
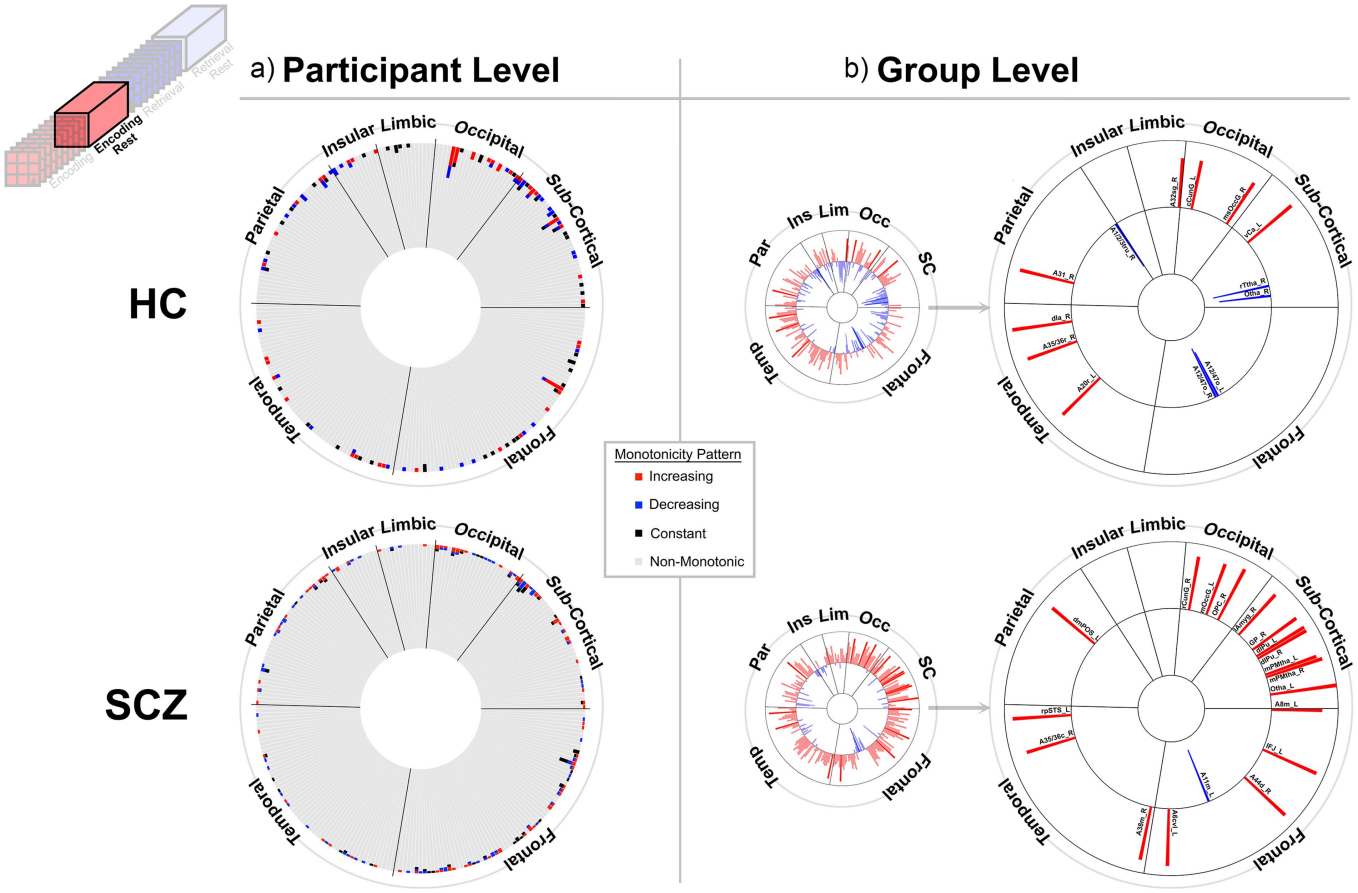
Monotonicity of BC_RO_ during Post-Encoding Rest. At the **(a)** participant and **(b)** group level. The presentation scheme is carried forward from Figure 3. **(a)** Again, in both groups, non-monotonicity was the norm (HC: 97.9%, SCZ: 98.2%). **(b)** Group level evidence (see Methods). More regions show positive monotonicity in SCZ (bottom), particularly within the subcortical nuclei with regional labels expanded on here. HC: A12/47o_L, Orbital Gyrus orbital area 12/47; A12/47o_R, Orbital Gyrus orbital area 12/47; A20r_L, Inferior Temporal Gyrus rostral area 20; dIa_R, Insular Gyrus dorsal agranular insula; A31_R, Precuneus area 31 (Lc1); A1/2/3tru_R, Postcentral Gyrus area1/2/3(trunk region); A32sg_R, Cingulate Gyrus sub genual area 32; cCunG_L, MedioVentral Occipital Cortex caudal cuneus gyrus; msOccG_R, lateral Occipital Cortex medial superior occipital gyrus; vCa_L, Basal Ganglia ventral caudate; rTtha_R, Thalamus rostral temporal thalamus; Otha_R, Thalamus occipital thalamus. SCZ: A8m_L, Superior Frontal Gyrus medial area 8; IFJ_L, Middle Frontal Gyrus inferior frontal junction; A11m_L, Orbital Gyrus medial area 11; A44d_R, Inferior Frontal Gyrus dorsal area 44; A6cvl_L, Precentral Gyrus caudal ventrolateral area 6; A38m_R, Superior Temporal Gyrus medial area 38; A35/36c_R, Parahippocampal Gyrus caudal area 35/36; rpSTS_L, rostroposterior superior temporal sulcus; dmPOS_L, Precuneus dorsomedial parietooccipital sulcus (PEr); rCunG_R, MedioVentral Occipital Cortex rostral cuneus gyrus; mOccG_L, lateral Occipital Cortex middle occipital gyrus; OPC_R, lateral Occipital Cortex occipital polar cortex; lAmyg_R, Lateral amygdala; GP_R, Basal Ganglia globus pallidus; dlPu_L, Basal Ganglia dorsolateral putamen; dlPu_R, Basal Ganglia dorsolateral putamen; mPMtha_L, Thalamus pre-motor thalamus; mPMtha_R, Thalamus pre-motor thalamus; Otha_L, Thalamus occipital thalamus.

**Figure 5:**
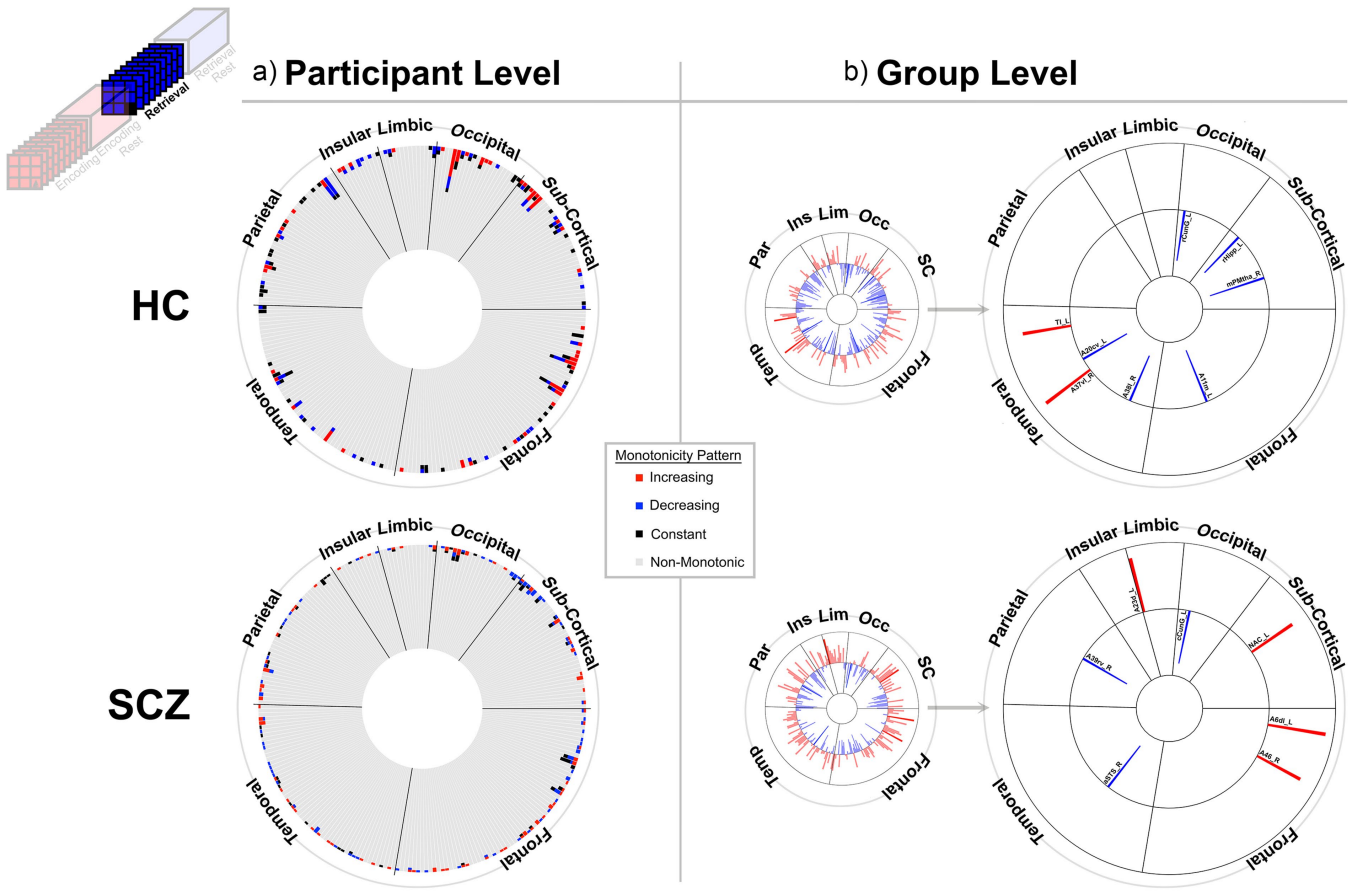
Monotonicity of BC_RO_ during Retrieval. The presentation scheme is carried forward from Figures 3, 4. **(a)** non-monotonicity remained the norm (HC: 96.9%, SCZ: 97.9%). **(b)** At the group level we list regions showing monotonicity. HC: A11m_L, Orbital Gyrus medial area 11; A38l_R, Superior Temporal Gyrus lateral area 38; A37vl_R, Inferior Temporal Gyrus ventrolateral area 37; A20cv_L, Inferior Temporal Gyrus caudoventral of area 20; TI_L, Parahippocampal Gyrus area TI (temporal agranular insular cortex); rCunG_L, MedioVentral Occipital Cortex rostral cuneus gyrus; rHipp_L, Rostral hippocampus; mPMtha_R, Pre-motor thalamus. SCZ: A6dl_L, Superior Frontal Gyrus dorsolateral area 6; A46_R, Middle Frontal Gyrus area 46; aSTS_R, Middle Temporal Gyrus anterior superior temporal sulcus; A39rv_R, Inferior Parietal Lobule rostroventral area 39(PGa); A23d_L, Cingulate Gyrus dorsal area 23; cCunG_L, MedioVentral Occipital Cortex caudal cuneus gyrus; NAC_L, Basal Ganglia nucleus accumbens.

**Figure 6:**
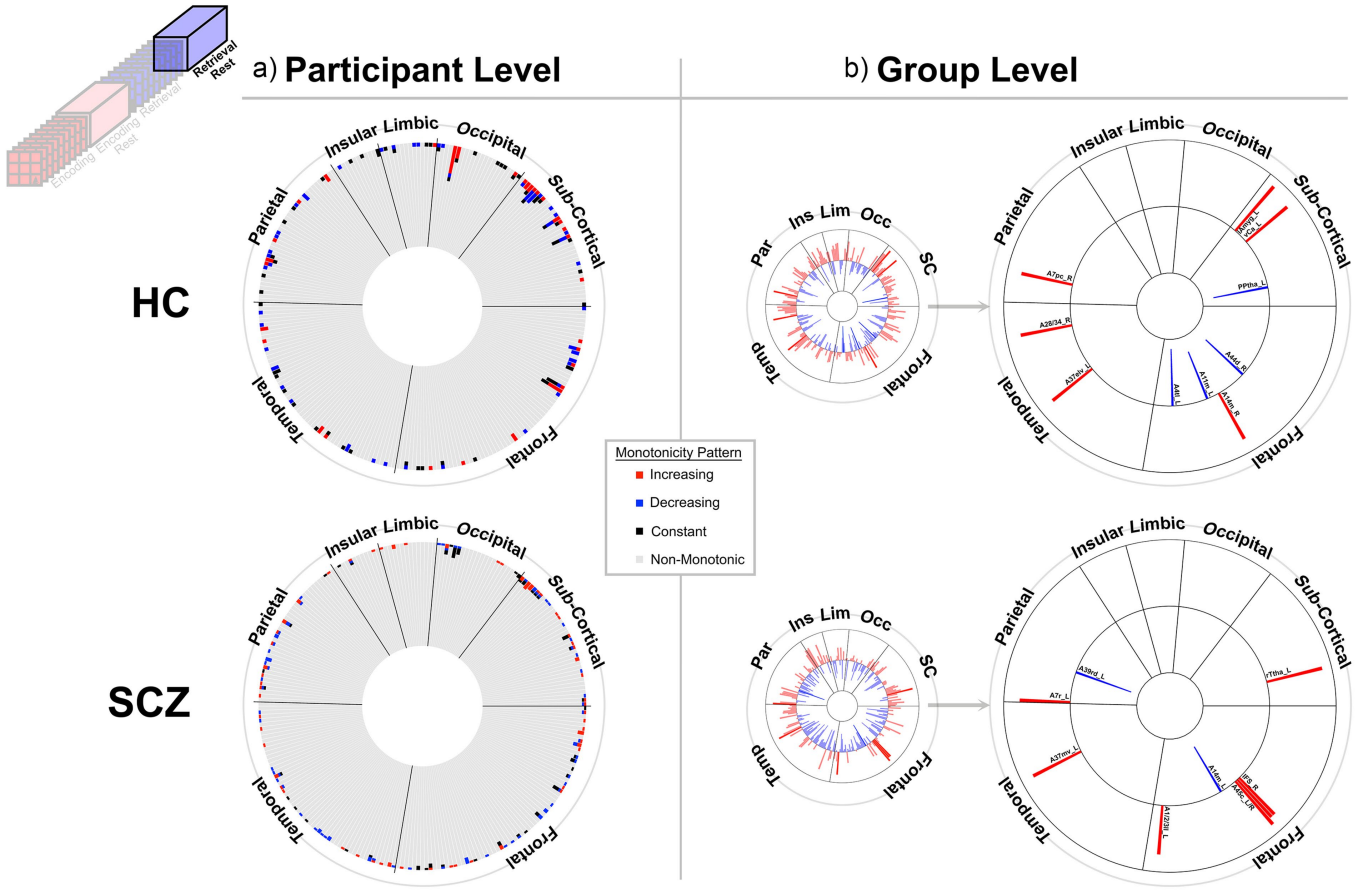
Monotonicity of BC_RO_ during Post-Retrieval Rest. The presentation scheme is carried forward from Figures 3–5. **(a)** At the participant level non-monotonicity of BC_RO_ remained the norm (HC: 97.8%, SCZ: 98.2%). **(b)** Both groups exhibited few but comparable numbers of regions displaying monotonic changes in BC_RO_. HC: A44d_R, Inferior Frontal Gyrus dorsal area 44; A14m_R, Orbital Gyrus medial area 14; A11m_L, Orbital Gyrus medial area 11; A4tl_L, Precentral Gyrus area 4(tongue/larynx region); A37elv_L, Inferior Temporal Gyrus extreme lateroventral area37; A28/34_R, Parahippocampal Gyrus area 28/34 (EC, entorhinal cortex); A7pc_R, Superior Parietal Lobule postcentral area 7; lAmyg_L, Lateral amygdala; vCa_L, Basal Ganglia ventral caudate; PPtha_L, Posterior parietal thalamus. SCZ: IFS_R, Inferior Frontal Gyrus inferior frontal sulcus; A45c_L, Inferior Frontal Gyrus caudal area 45; A45c_R, Inferior Frontal Gyrus caudal area 45; A1/2/3ll_L, Paracentral Lobule area1/2/3 (lower limb region); A37mv_L, Fusiform Gyrus medioventral area 37; A7r_L, Superior Parietal Lobule rostral area 7; A39rd_L, Inferior Parietal Lobule rostrodorsal area 39(Hip3); A14m_L, Orbital Gyrus medial area 14.

We observed a complex comport of results (behavioral and fMRI). In both groups, we replicated evidence for weak monotonicity in our behavioral data. This evidence was seen at the group level and frequently at the level of individual participants (Figure 2). In comparison, regardless of task condition, the functions formed from the graph theoretic summaries showed modest evidence for monotonicity (despite the large number of targets) (Figures 3–6). Trends at the group level were more reliable, but participant-level effects were sporadic. These results present an interesting trichotomy of effects: The behavioral results expectedly show evidence of monotonic changes with time. However, there was muted evidence for such effects in graph theoretic summaries of both the group and individual participant level fMRI data. Our findings underscore the challenge of linking overt behavioral measures to fMRI measures of brain function, even when (a) the fMRI data are co-acquired with the behavior and (b) are interrogated using well-established principles like monotonicity.

## 2 Methods

### 2.1 Participants

Data (behavioral and fMRI) were collected from eighty-eight participants. Healthy controls (HC, *N* = 39) were recruited using community advertisements and flyers, and schizophrenia patients (SCZ, *N* = 49) were recruited from out-patient clinics run by the Wayne State University Physician’s Group. All methods were approved by the Institutional Review Board (IRB) at Wayne State University and participants provided informed consent to participate. By definition, HC participants were free of any psychiatric diagnosis, while SCZ participants met DSM-5 criteria for schizophrenia (patients were evaluated by treating physicians and a licensed psychologist, UR). Patients were stabilized on their prescribed regime of antipsychotics (Full demographic information is provided in Table 1).

**Table 1:** The demographics (patients and controls) and clinical characteristics (of patients) are presented.

	Healthy controls	Schizophrenia patients
*N*	39	49
Age (years)	27.59 ± 6.71	31.47 ± 8.61
Gender	10 F/29 M	9 F/40 M
Handedness	30 Right	37 Right
FSIQ-4 score	101.85 ± 9.46	88.29 ± 7.94
PANSS positive score		13.76 ± 3.44
PANSS negative score		14.24 ± 3.92
PANSS general score		26.02 ± 5.81

All patients were stabilized using atypical antipsychotics.

### 2.2 Magnetic resonance imaging

Magnetic resonance (MR) data were acquired on a 3 T Siemens Verio scanner with a 32-channel head coil at WSU’s Vaitkevicius Imaging Center located in Harper Hospital. During MRI, participants’ heads were stabilized using foam inserts, and ear plugs were used to minimize the intrusive effects of scanner noise. All participants completed the MR procedures without incident. fMRI data were collected using a multiband gradient EPI fMRI sequence (TR = 3 s, TE = 24.6 ms, multiband factor = 3, FOV = 192×192 mm^2^, matrix = 96×96, 64 axial slices, voxel resolution = 2 mm^3^). In addition, a high-resolution T_1_-weighted anatomical image was also collected for normalization and co-registration with the EPI scan (3D Magnetization Prepared Rapid Gradient Echo Sequence, TR = 2,150 ms, TE = 3.5 ms, TI = 1,100 ms, flip angle = 8 degrees, FOV = 256x256x160 mm^3^, 160 axial slices, pixel resolution = 1 mm^3^).

### 2.3 The associative learning paradigm

fMRI data were co-acquired while participants learned nine object-location (in a two-dimensional grid) associations using a previously established paradigm (Diwadkar et al., 2008; Brambilla et al., 2011; Samona et al., 2024). Figure 1 shows the paradigm structure. Each of the eight successive iterations consisted of sequential epochs (27 s each) for Encoding, Post-Encoding Rest, (Cued) Retrieval and Post-Retrieval Rest. During Encoding, each of nine equi-familiar objects was presented in its associated locations (squares within a 3×3 grid) for naming (3 s/object). Participants were shown a fixation marker during each Post-Encoding Rest epoch. Next, learning proficiency was tested during Retrieval epochs; here, locations were cued in random order, and participants were required to verbalize the name of the object associated with each cued location (no feedback was provided). Responses were recorded through the built-in microphone relay and scored for correctness. Retrieval was the only condition during which overt behavior was recorded. The Post-Retrieval and the Post-Encoding Rest epochs were identical.

### 2.4 fMRI data preprocessing

fMRI data were preprocessed using established methods for temporal (slice timing correction) and spatial correction (realignment and normalization) (SPM12, https://www.fil.ion.ucl.ac.uk/spm/). The EPI images were manually oriented to the AC-PC line with the reorientation vector applied across the image set. The images were then realigned to a reference image (to correct for and quantify head motion), and co-registered to the anatomical high-resolution T_1_ image. The T_1_ image was normalized to the Montreal Neurological Institute (MNI) template, and the resultant deformations were applied to the co-registered EPI images. Low-frequency components were removed (1/128 filter), and the images were resliced (2 mm^3^) and smoothed (8 mm FWHM). In first-level models, task epochs were modeled as boxcar functions and convolved with a canonical hemodynamic response function to form the regressors of interest. In each participant’s first-level model, the six motion parameters (three each for translation and rotation) derived from realignment were modeled as covariates of no interest. In any participant, images exceeding 4 mm of movement (<1% of all images) were excised from that participant’s data without replacement (motion estimates recovered from reconstruction did not differ between groups, *p*’s > 0.1). This approach to image processing and modeling has been consistently applied in fMRI studies of clinical and non-clinical populations (Ravishankar et al., 2019; Baajour et al., 2020; Muzik et al., 2020; Meram et al., 2021; Tso et al., 2021; Muzik et al., 2022; Zovetti et al., 2022; Hasan et al., 2023; Samona et al., 2024).

### 2.5 Estimating undirected weighted graphs

All subsequent analyses were conducted using specially developed scripts in *R* (R_Core_Team, 2021) (scripts are available in the GitHub link) and MATLAB (MathWorks, 2007). From the processed fMRI data, signals were averaged across voxels in each of 246 parcels in the Brainnetome atlas (Fan et al., 2016; Li et al., 2019). This atlas was chosen based on its multi-modal and multi-task constitution (Fan et al., 2016), and because it provides excellent functional parcellation of the cerebrum. 246 unique time series (each of length 288 points) were formed from each participant’s data. All subsequent analyses were conducted on these 288-point time series.

Each time series captures changes evoked in that region across the eight task iterations. In each participant, and in each of the four conditions (Encoding, Post-Encoding Rest, Retrieval. Post-Retrieval Rest), we computed the full undirected functional connectivity (uFC) matrix (based on Fisher Z transformed stationary zero-lag Pearson’s correlation) (Silverstein et al., 2016; Thompson and Fransson, 2016) for each of the eight iterations. Thus, each matrix captures the uFC between 30,135 unique pairs (_246_*C*_2_), and is equivalent to a weighted undirected graph with 246 vertices and 30,135 edges (Newman, 2004). Each *full* weighted graph was carried forward for graph theoretic analyses. We maintained full (as opposed to thresholded) graphs as this allowed us to (a) preserve the complete distribution of connectivity strengths and (b) avoid inequitable (across participants) effects of edge thresholding. While edges with low weights are likelier to represent noisy interactions that could be discounted (van Wijk et al., 2010; Richiardi et al., 2011), edge removal can impact the integrity of graph theoretic measures that are fundamentally designed to operate on fully connected and weighted networks, and which are known to provide reliable insights even when the graphs retain weak connections (Rubinov and Sporns, 2011). Moreover, studies indicate that removing such edges has minimal effects on significance (Civier et al., 2019).

### 2.6 Estimating BC and BC rank order

BC is an estimate of the number of shortest functional paths traversing through a vertex of a graph where (in our case) the uFC metric represents functional “distance.” In our implementation, higher edge weights (i.e., stronger functional connections) were treated as shorter path lengths. BC was computed as follows (Equation 1).


(1) $$BC^{\text{weighted}}=\frac{1}{\left(n-1)(n-2\right)}\Sigma_{h,j\neq i}\frac{sp_{\mathrm{hj}}(i)}{sp_{\mathrm{hj}}}$$

The expression *sp_hj_(i)* represents the number of shortest paths between vertices *h* and *j* that passes through vertex *i*. In the context of fMRI, BC appears to represent a region’s role in transmitting and facilitating interactions across the connectome (Rubinov and Sporns, 2010; van den Heuvel et al., 2010). More generally, nodes in any functional connectome can be ordered by BC (highest to lowest, or vice versa), where the resultant ordinal ranking represents the node’s relative importance within the network (Sporns, 2014; Meram et al., 2023). Accordingly, for each undirected weighed graph (estimated across one 27 s task condition iteration), each of the 246 nodes was assigned a BC rank order (BC_RO_) value (between 1 and 246).

Betweenness Centrality (BC) was ideal for our purposes because as our primary hubness metric, it characterizes a node’s contribution to global communication and integration across the entire network (Freeman, 1977) and quantifies the total weighted length of all shortest paths that pass through any node. This makes it particularly suitable for assessing functional network configurations during learning, and for investigating whether these undergo monotonic changes over the task. This choice fundamentally aligns with our interest in understanding whether monotonicity is a global principle that might emerge from distributed network interactions rather than from localized activations. For our purposes, rank order was a more meaningful measure than the actual BC values, because the latter are a function of the estimated connectivity in the adjacency matrix (Freeman, 1977), which itself can depend on several factors across the experiment. By focusing on BC_RO_ we could conduct investigations on whether the relative importance of a node within each epoch changes over the course of the task.

### 2.7 Estimating ASPL (average shortest path length)

ASPL, is a measure of network efficiency, and is conventionally defined as the average number of edges along the shortest paths for all possible pairs of nodes in any network. In fMRI-based connectomics, ASPL constitutes a measure of global integration across the connectome, where smaller ASPL reflects a functionally more efficient network (Betzel et al., 2016). ASPL was computed as follows (Equation 2):


(2) $$\text{ASPL}=\frac{1}{n(n-1)}\sum_{i1j}d(v_{i},v_{j})$$

where computed edge weights (Fisher’s *Z* values derived from Pearson *R* values) were inverted to re-represent the edges as shortest paths [permitting us then to use Dijkstra’s algorithm (West, 2001)].

### 2.8 Assessing weak monotonicity

In any class of data (*D*), where *D_n_* represents the value at time *n,* weak monotonicity across a trend is satisfied if:


$$a)\;D_{1}\leq D_{2}\leq \ldots \leq D_{8}\;\text{or}\;\;b)\;D_{1}\geq D_{2}\geq \ldots \geq D_{8},\text{where}\;D_{1}\neq D_{8}$$

We used a more liberal version of weak monotonicity, in which we permitted one violation of any trend (e.g., *D*_1_ ≤ *D*_2_ ≤ *D*_3_ > *D*_4_ ≤ D_5_ ≤ D_6_ ≤ D_7_ ≤ D_8_).

Weak monotonicity was investigated in each of the behavioral and imaging measures across the eight repetitions of the task. For the behavioral measures, the variable of interest was the proportion of correct responses in each of the eight Retrieval epochs. For BC, we investigated weak monotonicity for *each node*’s BC_RO,_ separately over eight iterations of each of the four task conditions. Finally, for ASPL, we investigated weak monotonicity at the level of the functional connectome, with ASPL itself being the variable of interest. Here we used a three-way mixed analysis of variance (ANOVA) which allowed us to assess the effects of Task Condition (non-independent factor), Time (non-independent factor), and Group (independent factor). Any evidence of monotonicity would be revealed in a main effect of time (with a significant linear contrast), while this statistical approach also allowed us to investigate main effects for Group and Condition (and all two- and three-way interactions). We also assessed weak monotonicity at the *group level*. Here, in each of the HC and SCZ groups, BC_RO_ values for each of the 246 nodes were *averaged (across participants)* for each condition and iteration of the task and assessed for weak monotonicity using Spearman’s ρ between iteration number (1–8) and the group-averaged BC_RO_ values (Spearman, 1987) (*p* < 0.05). For ASPL, monotonicity was assessed across each condition with group-averaged data using the weak monotonicity test (see above).

### 2.9 Supplementary analyses

In supplementary analyses, we investigated monotonic changes *in the fMRI signal* in each participant. For every region in every participant, fMRI signal amplitude for every image (time point) was expressed as percent change over the average signal of that region across the entire acquisition. We then assessed monotonicity for the signal amplitude for each task condition across the eight iterations of the task (see Supplementary Figures 2–5). We also assessed the distribution of edge weights, defined as Fisher z transformed Pearson correlation coefficients from the uFC matrices, across all eight iterations for each task condition, separately for HC and SCZ (Supplementary Figure 6). The Appendix provides an overview of our approach toward statistical inference.

## 3 Results

**Figure 7:**
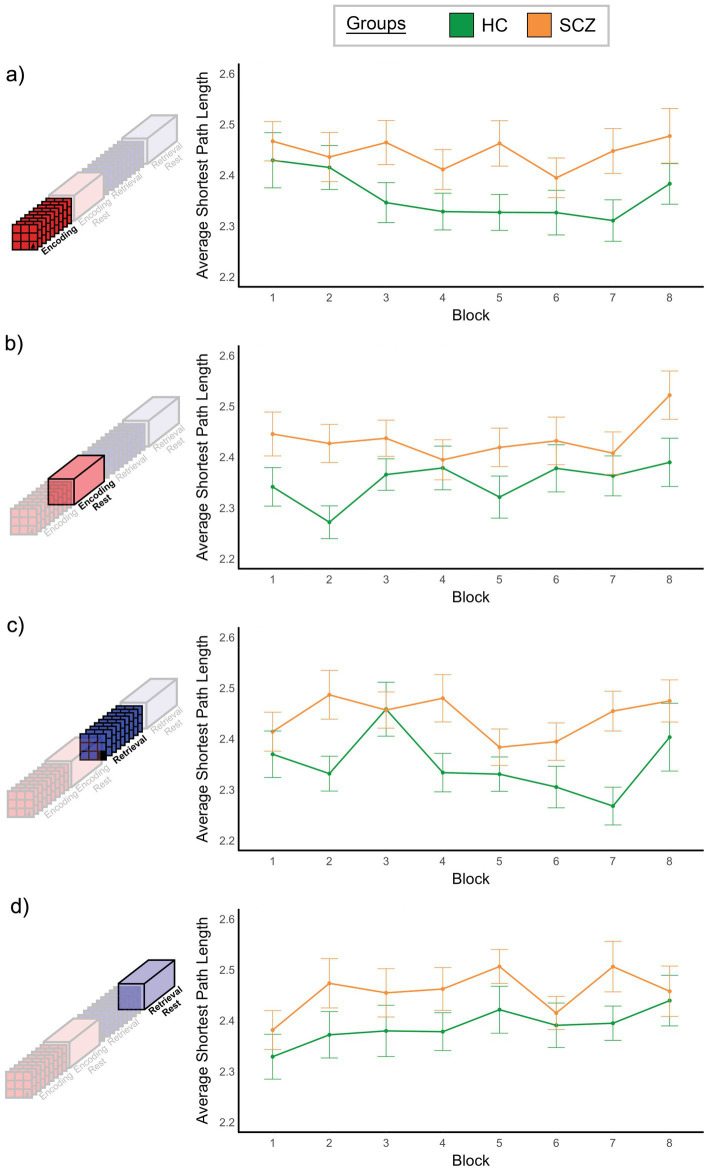
Monotonicity of ASPL. For both groups mean ASPL is plotted over the eight repetitions of each task condition. We did not observe a significant effect of Time or interactions that involved Time (see Results). However, applying the weak monotonicity test (see Section 2.8), the Encoding condition in HC exhibited weakly monotonic behavior.

The results are staged as follows: (1) First, we present behavioral data from participants in both groups, and provide group averaged behavioral functions (Figure 2). These data show that weak monotonicity was satisfied in both the group averaged data and in data from a preponderance of participants. (2) Next, for the BC_RO_ data, we provide assessments of monotonicity in individual participants *and* group averaged investigations for each of the four task conditions (Figures 3–6). (3) The search for monotonicity in the ASPL data is reproduced next (Figure 7).

### 3.1 Monotonicity in behavior: task proficiency

Figure 2 provides information on (a) the frequency with which weak monotonicity was observed across individual participants in each group (Figure 2a), and (b) group averaged functions (Figure 2b). In ~90% of HC participants and in ~60% of SCZ patients, behavioral proficiency increased monotonically (red portion of the stacked bars), though the proportion was greater in healthy controls than in patients (Fisher’s Exact test, *p* < 0.05) (Freeman and Campbell, 2007). The group averaged data satisfied the weak monotonicity test in both groups (Figure 2b) (the smoothed functions were fitted using LOESS, *λ* = 1.26 × 10^−5^) (Jacoby, 2000).

### 3.2 Monotonicity in BC

Figures 3–6 provide evidence for any observed monotonicity in BC_RO_. Data are presented separately for the participant level (a) and the group level (b) analysis. Each figure represents results from one of the four task conditions. In each sub-figure (a), the 246 brain regions (nodes) are depicted in a circular arrangement and are grouped by lobe (regional names are withheld to reduce clutter). The stacked frequency plots radiate inwards, and here, each bar represents the frequency of participants who showed varying patterns of monotonicity (red or blue) or non-monotonicity (black or gray) for any specific node (see color bar in the center of the figure). Across Figures 3a–6a, we observed monotonicity in BC_RO_ in a small number of nodes (see Appendix).

The adjoining group analyses (Figures 3b–6b) complement analyses in individual participants (Figures 3a–6a). In these sub-figures, each bar in the smaller circles represents the Spearman’s ρ for that region and group (red and blue bars, respectively, represent positive and negative ρ values). Spearman’s ρ was used on this rank ordered data because it effectively is a measure of monotonicity: perfectly monotonic functions have ρ of (−/+)1, while trends toward monotonicity result in values of higher magnitude, and non-monotonic trends converge on 0. Opaque (as opposed to translucent) bars denote significant effects (*p* < 0.05). Only significant effects are migrated over to the larger circles (with region names added).

At least two effects are noteworthy: First, the observed trends in the group level data partially complemented those from the individual participant data, in that each analyses revealed different brain regions (noticeable when comparing across (a) and (b) in each figure). These differences, while difficult to parse are consistent with studies that have focused on other fMRI metrics. Individual variability is a pervasive property (or perhaps “nuisance”) of fMRI signals (Van Horn et al., 2008) and has motivated the need to quantify individual differences in activation and connectivity data (Dubois and Adolphs, 2016; Finn and Rosenberg, 2021; Catanzaro et al., 2024), as well as the repeated imaging of individual subjects in order to quantify intra-subject variability (Gountouna et al., 2010; Gorgolewski et al., 2013). Second, across the eight analyses (four conditions and two groups), somewhat discernable inter-group differences in monotonicity were observed (though primarily at the group level), primarily for the Encoding epochs (Figure 3b) and Post-Encoding Rest epochs (Figure 4b). In the SCZ group a substantial number of cortical and sub-cortical nodes (14 and 18 respectively) displayed significant monotonic changes, though this was less the case for the control group. For instance, during Encoding (Figure 3b), monotonicity was observed most notably in middle frontal, cingulate and medial temporal regions like the parahippocampal gyrus, regions that have all been previously linked with associative learning and memory (Suzuki, 2008; Baajour et al., 2020; Hasan et al., 2023). These inter-group differences (where more substantial monotonicity was observed in patients) might relate to differences in the dynamic expression of task demands. Thus, over the course of learning, schizophrenia patients may need an *increase* in attention-related control mechanisms to fulfill the demands of the task (Le Pelley et al., 2016), and this need may be expressed in an increase in the integrative importance of brain regions. Such an explanation could also be evoked to explain the relatively heavy representation of sub-cortical nodes during Post-Encoding Rest (Figure 4b). The Post-Encoding Rest period is one in which previously shown associations are covertly recapitulated and rehearsed (see Figure 1), and may involve the re-engagement of cognitive and perceptual interactions that are characteristic of depictive mental imagery (Kosslyn et al., 1995; Pearson and Kosslyn, 2015). This may explain why the integrative importance of sub-cortical regions in schizophrenia increases over the timeline of the task. Of course, these interpretations are fundamentally speculative, cannot confirm the precise nature of regional functions and are subject to many of the challenges of “reverse inference” (Sprooten et al., 2017; Diwadkar and Eickhoff, 2021). However, results like these suggest that a framework like monotonicity might be profitably used to constrain evaluation of changes in brain network function over some task period.

### 3.3 Monotonicity in ASPL

The results of the three-way ANOVA (see Section 2.8) did not reveal a significant effect of Time, *F*_7, 602_ = 1.794, *p* > 0.05, or significant interactions involving Time (x Group, x Condition, x Group x Condition, all *p*’s > 0.1). Figure 7 depicts the relationship between time (horizontal axis) and ASPL (vertical axis) in each of the four conditions, confirming an absence of monotonic changes in ASPL. The only significant effect that we observed was a significant effect of group, *F*_1, 86_ = 11.47, *p* < 0.001. Here, ASPL was significantly smaller in HC compared to SCZ (see Supplementary Figure 1). This effect underlines the inefficiency of brain network function in schizophrenia (Zovetti et al., 2022), and is consistent with independent task- and resting-fMRI studies that show increased ASPL in schizophrenia (van den Heuvel et al., 2010; Rubinov and Bassett, 2011).

### 3.4 Monotonicity in regional fMRI signal change

In supplementary analyses (Supplementary Figures 2–5), we assessed changes in the fMRI signal in each of the 246 regions for evidence of monotonicity. These analyses complement the graph theoretic work at the individual participant level (Figures 3a–6a) by providing a narrower focus on the responses of *individual* regions (rather than the integrative those regions’ integrative role). The results provided sporadic evidence for monotonicity in several participants, particularly in frontal regions (the highest frequency was of HC participants in the inferior frontal gyrus during the Encoding-Rest condition, Supplementary Figure 3a). However, in general no clearly discernable inter-group differences were seen, further underlining the complementary nature of the two types of analyses.

## 4 Discussion

Our study’s motivations were straightforward: Do behavioral and fMRI data co-acquired during associative learning evince similar patterns of monotonicity? In each of four task conditions, and across eight iterations of the task, we calculated regional (Rubinov and Sporns, 2010) and global (Betzel et al., 2016) graph theoretic measures, before both were tested for monotonic changes (Figures 3–7). This approach was replicated for fMRI signal change data (Supplementary Figures 2–5) (Logothetis and Wandell, 2004). Furthermore, we investigated monotonicity at the level of individual participants *and* in group averaged data. Finally, we also tested for monotonicity in data acquired in both healthy controls and in schizophrenia.

First, analyses of overt behavior (Figure 2) replicated classic negatively accelerated learning (Balsam et al., 2010), effects that have also been occasionally reported at the neuronal level (Okada, 1996; Wirth et al., 2003). Second, there was partial evidence for monotonicity in fMRI measures for both local and global graph theoretic measures, as well as the signal change data.

### 4.1 Monotonicity in brain, behavior and psychology

In mathematics, monotonic functions preserve the mapping (forward or inverse) between ordered sets (Merkle, 2014). More generally, monotonicity loosely characterizes the relationship between psychological responses to graded changes in stimulus characteristics. For instance, when human participants are asked to discriminate between physical magnitudes intervals, their judgments generally preserve the relative order between those magnitudes (Gliksman et al., 2016). This evidence conforms to Steven’s power law which states that sensation magnitudes grow as power functions of the stimulus intensities that produce these sensations (Stevens, 1957; Zwislocki, 2009). Thus, in psychophysical experiments, changes in stimulus magnitude and the concomitant changes in psychological responses (or sensations) form ordered sets that preserve a monotonic mapping. Such mappings have been documented in diverse phenomena ranging from nociceptive responses to changes in pain stimulation (Arndt and Klement, 1991), working memory load (Baddeley, 1986) and appetitive judgments to the complexity of visual stimuli (Aitken, 1974). As noted, animal studies have shown that neurons sometimes evince monotonic responses to the magnitude of reward (in the amygdala) (Bermudez and Schultz, 2010), and the numerosity of visual elements (intra-parietal sulcus) (Roitman et al., 2007). These (and other) studies suggest that monotonicity may be a central principle linking psychology, biology and mathematics (Grice et al., 2023). In higher order domains like learning (and especially associative learning), the ordered sets of (a) the time steps (over which learning accrues) and (b) behavioral proficiency (how proficient learning is) are invariably yoked in monotonic relationships (see Figure 2). This may partly be because reward expectation (which is central to studies of animal learning) is monotonically related to the conditioned response (Gershman, 2015), and the *uncertainty* associated with learning *decreases* as a function of the time horizon over which learning occurs (Muzik and Diwadkar, 2023). Finally, learning traces in the hippocampus also accumulate in a non-linear but generally monotonic manner (Norman and O'Reilly, 2003; Diwadkar et al., 2008).

The conditions under which monotonicity in behavior is *co-observed* with monotonicity in fMRI measures are somewhat unclear. To our knowledge, the closest evidence comes from two sources: (a) Using an event-related fMRI study of paired-associate memory (which is somewhat different from the class of learning implemented in this investigation) (Law et al., 2005), Law and colleagues demonstrated that fMRI responses in medial temporal lobe regions like the hippocampus, parahippocampus and the perirhinal cortex increase monotonically with memory strength and (b) In two separate effective connectivity studies (Friston, 2009), Büchel et al. (1999) and Banyai et al. (2011) showed that the effectivity connectivity of frontal-hippocampal pathways increased over the course of learning. However, these studies and others (Toni et al., 2001) did not use graph theoretic measures and also did not test for monotonicity in fMRI data in *individual* participants.

### 4.2 Graph theoretic summaries of fMRI data

Graph theoretic measures efficiently summarize spatio-temporal fMRI data (and indeed any data) (van den Heuvel and Sporns, 2013; Farahani et al., 2019; Meram et al., 2023) and by doing so, reveal hidden structure in any complex system. Measures like BC are sensitive to the integrative importance of a node because BC quantifies the notion of “hubness”; this is the degree to which a node acts as a *bridge* along the shortest path between *any two other* nodes (Koschützki et al., 2005). Variations in BC capture functional network dynamics at the nodal level. In other words, changes in a node’s hubness encode changes in the functional properties of a network (de Pasquale et al., 2018; Meram et al., 2023). Thus, it is precisely because behavior is buttressed by network interactions, that we should expect to observe monotonic changes in behavioral proficiency in conjunction with some monotonic changes in network properties (Ravishankar et al., 2019; Samona et al., 2024).

Interestingly, such evidence was observed when analyzing data from individual participants (Figures 3a–6a) but was relatively sparse (given the large corpus of potential targets). It may be that fMRI signals are highly variable in individual participants, and this variability affects summative graph theoretic measure as well (Falco et al., 2019; Falco et al., 2020). However, and unsurprisingly, group level summaries were more promising. Here, the most explainable effects were observed in the *SCZ group* during Encoding (Figure 3b, bottom). Given the frontal-temporal basis of learning (Davachi and Wagner, 2002; Ranganath et al., 2004; Schlichting and Preston, 2016; Woodcock et al., 2016), evidence of the monotonicity of BC in multiple frontal (medial, inferior and orbital) nodes, and nodes in the temporal lobe (most notably parahippocampal) is compelling. On average, in SCZ the hubness of these nodes increased monotonically over time. We also observed an eloquent set of findings during the Post-Encoding Rest condition (which has been noted for being a constructive “passive” state) (Schlichting and Preston, 2014; Samona et al., 2024). Here, patients showed increases in the hubness of sub-cortical nodes, most notably in thalamic sub-regions, and nodes in the occipital lobe (Figure 4b, bottom).

Both BC and ASPL are based on considering the shortest paths between nodes, but ASPL *summarizes all paths* into a single measure of network efficiency and is therefore presumed to complement BC. Our analysis appears to corroborate this fact because SCZ showed significantly *higher* ASPL values (Supplementary Figure 1). This increase has been associated with inefficient network communication, an established feature of the schizophrenia brain (Rubinov and Bassett, 2011; Su et al., 2015; Zovetti et al., 2022). Indeed, in healthy controls, ASPL decreased monotonically across the eight repetitions of Encoding (see Figure 7a). This decrease is evidence for associations being successfully consolidated as the underlying network interactions in the healthy brain become *more efficient*. Unsurprisingly, these changes in and differences in ASPL are likely to be related to the overall connectivity in a network, particularly in fully weighted networks where *increased connectivity* is typically associated with *shorter average path length* (Perez and Germon, 2016). In supplementary analysis, we confirmed this to be true in our data. Supplementary Figure 6 provides an inter-group comparison in the distribution of correlation coefficients across all four conditions, where we observed a leftward shift in the schizophrenia data, thus confirming that ASPL is a measure of cumulative inefficiency (or efficiency in a network).

## 5 Limitations and conclusions

Many decisions can influence the kinds of inferences about brain networks drawn from fMRI data. These factors include decisions about how nodes are spatially defined in order to form a network of interest (Eickhoff et al., 2018; Falco et al., 2019; Falco et al., 2020), the choices made with respect to data filtering and denoising (Andronache et al., 2013) and the width of smoothing filters used in preprocessing (Alakorkko et al., 2017; Triana et al., 2020). While, the few studies on these issues indicate that the exercised choices do not introduce *systematic* bias in observed results, this is an admitted limitation of our analyses, where our choice of template (Fan et al., 2016) and the parameters in the preprocessing steps can have impacts on our results.

Next, our motivations might appear to be suited for the use of dynamic functional connectivity, a technique of choice for studying task-evoked or resting-state dynamics in fMRI signals (Hutchison et al., 2013; Heitmann and Breakspear, 2018). However, we were specifically motivated to treat each epoch as “discrete” and separate “events” on the path toward learning (whereas by definition, dynamic functional connectivity operates along moving and overlapping windows of time within a task). Doing so was the only way to assess whether across the task *both* behavioral and fMRI related functions displayed evidence for monotonicity. Clearly dynamic functional connectivity has a crucial role to play in understanding the dynamics of learning (Fatima et al., 2016), and is an endeavor of some of our ongoing work (Bhatt et al., 2025).

What is the relationship between network metrics and overt human behavior? For several reasons this remains a vexing question in human neuroscience; (i) the relatively obscure relationship between neurophysiological signals at different spatio-temporal scales makes it difficult to model the manner in which signals converge or diverge across the cortical hierarchy (Singh, 2012); (ii) brain network interactions (which may be the most proximate physiological correlate of behavior) are inherently probabilistic (as opposed to deterministic) and are therefore challenging to capture using formal computational frameworks (Mannino and Bressler, 2015; Razi and Friston, 2016); (iii) outside of basic behaviors (like reflex arcs etc.) no models straightforwardly explain how interactions in the biological substrate translate into overt behavior (Krakauer et al., 2017). We do not expect a single study to adequately address all these questions. However, we hoped to contribute to this dialectic. Therefore, we motivated a specific attempt to co-observe monotonicity in behavior, *and* in connectomic and regional measures of fMRI data across two comparator groups. Our investigation attempted to be as comprehensive as possible, using multiple outcome measures, clear definitions of monotonicity, and the use of a healthy and a clinical group. We were able to modestly co-observe monotonicity (more so at the group level) in behavioral and connectomic measures. This evidence is interesting because (as stated) no straightforward mechanistic framework links the diverse mix of fMRI signals to overt behavior; as has been repeatedly noted, behavior is *unlikely* to emerge from one-to-one mappings to specific neural events (Price and Friston, 2005). Rather, psychological events may emerge from distributed activity across neural ensembles rather than local activity in circumscribed neural populations, thus leading to a labyrinthine relationship between behavior and its underlying neurophysiological correlates (Westlin et al., 2023). Countless theoretical overviews emphasize these points (Tranel, 2007; Park and Friston, 2013) and suggest that the relationship between overt behavior and measures of brain function is going to be complex. These challenges *may* be overcome through the simultaneous modeling of multiple signal sources (including EEG) (Turner et al., 2016). Nevertheless, our reliance on a well-defined mathematical principle like monotonicity may provide one approach toward making a dent in this challenge (for example, other such efforts in fields like mathematical psychology have proven insightful in elucidating the perceptual bases of the sensorial world, or how similarities in object features are represented along internal psychological dimensions) (Tversky, 1977; Shepard, 2001). If two classes of co-acquired signals (in our case, behavior and fMRI data) display similar temporal forms (i.e., similar monotonic changes), then one might infer that they are related in a meaningful way. Many such functionalist approaches are not predicated on a clear *mechanistic* understanding of how the two sets of signals emerge from each other (Shoemaker, 1981), but rather on the roles that the play in a process. Thus (and as stated in the Introduction), monotonicity can be a mathematical anchor, and we have used it to unearth modest evidence linking behavior to fMRI data. Future studies will be needed to better explain the limits of our approach, and to confirm or reject its validity.

## Data Availability

The datasets presented in this study can be found in online repositories. The names of the repository/repositories and accession number(s) can be found at: NIH Public Repository indexed by Grant # MH117777. https://github.com/Dbhatt1/Monotonicity-in-graph-theoretic-summaries-of-fMRI-data-acquired-during-human-learning-.

## Supplementary material

SUPPLEMENTARY FIGURE 1 ASPL of HC and SCZ across all conditions and blocks. A three-way ANOVA (Group, Time and Condition) revealed a main effect of group, with SCZ showing significantly higher ASPL (*F*_1,86_ = 11.47, *p*<0.001, *MSE* = 0.187) indicative of a loss of network efficiency.

SUPPLEMENTARY FIGURE 2 Monotonicity of fMRI signal amplitudes during Encoding at the participant level for **(a)** HC and **(b)** SCZ. The figure complements Figure 3a and the arrangement is maintained. As was the case with the findings from BC_RO_, non-monotonicity was the norm in a majority of participants. This sparsity is also seen in Supplementary Figures 3–5.

SUPPLEMENTARY FIGURE 3 Monotonicity of fMRI signal amplitudes during Post-Encoding Rest at the participant level for **(a)** HC and **(b)** SCZ. The figure complements Figure 4a.

SUPPLEMENTARY FIGURE 4 Monotonicity of fMRI signal amplitudes during Retrieval at the participant level for **(a)** HC and **(b)** SCZ. The figure complements Figure 5a.

SUPPLEMENTARY FIGURE 5 Monotonicity of fMRI signal amplitudes during Post-Retrieval Rest at the participant level for **(a)** HC and **(b)** SCZ. The figure complements Figure 6a.

SUPPLEMENTARY FIGURE 6 Distributions of edge-weight (Fisher z transformed correlation coefficients derived from the uFC matrices) across the eight iterations for each task condition in HC and SCZ.
