# Supplementary figures and images for: Monotonicity in graph theoretic summaries of fMRI data acquired during human learning
Source: Front Hum Neurosci. 2025 Sep 22;19:1595331. doi: 10.3389/fnhum.2025.1595331 (PMC12497747; doi:10.3389/fnhum.2025.1595331)

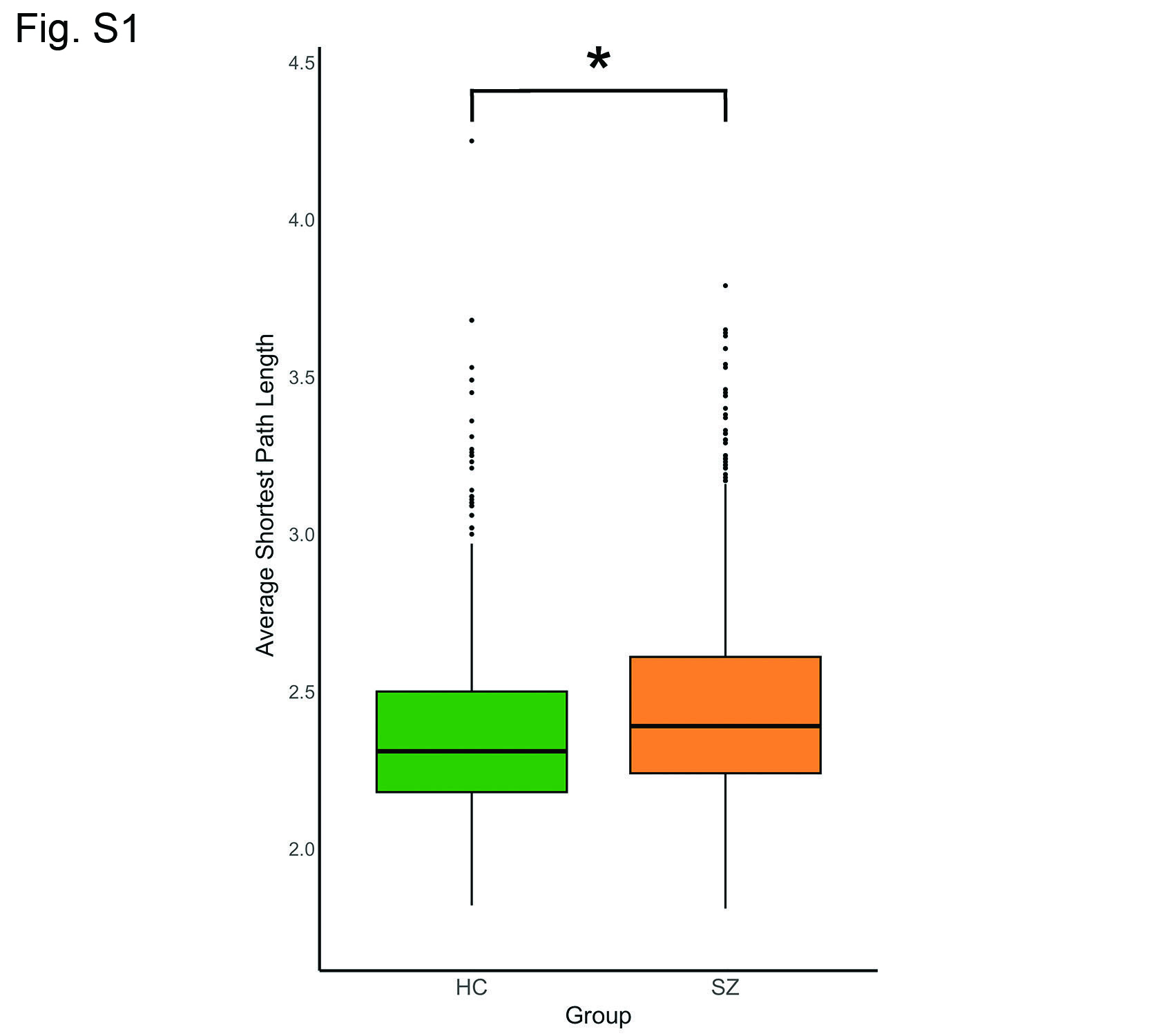

Supplement: SUPPLEMENTARY FIGURE 1 — ASPL of HC and SCZ across all conditions and blocks. A three-way ANOVA (Group, Time and Condition) revealed a main effect of group, with SCZ showing significantly higher ASPL (F1,86 = 11.47, p<0.001, MSE = 0.187) indicative of a loss of network efficiency. [file Image_1.jpeg]

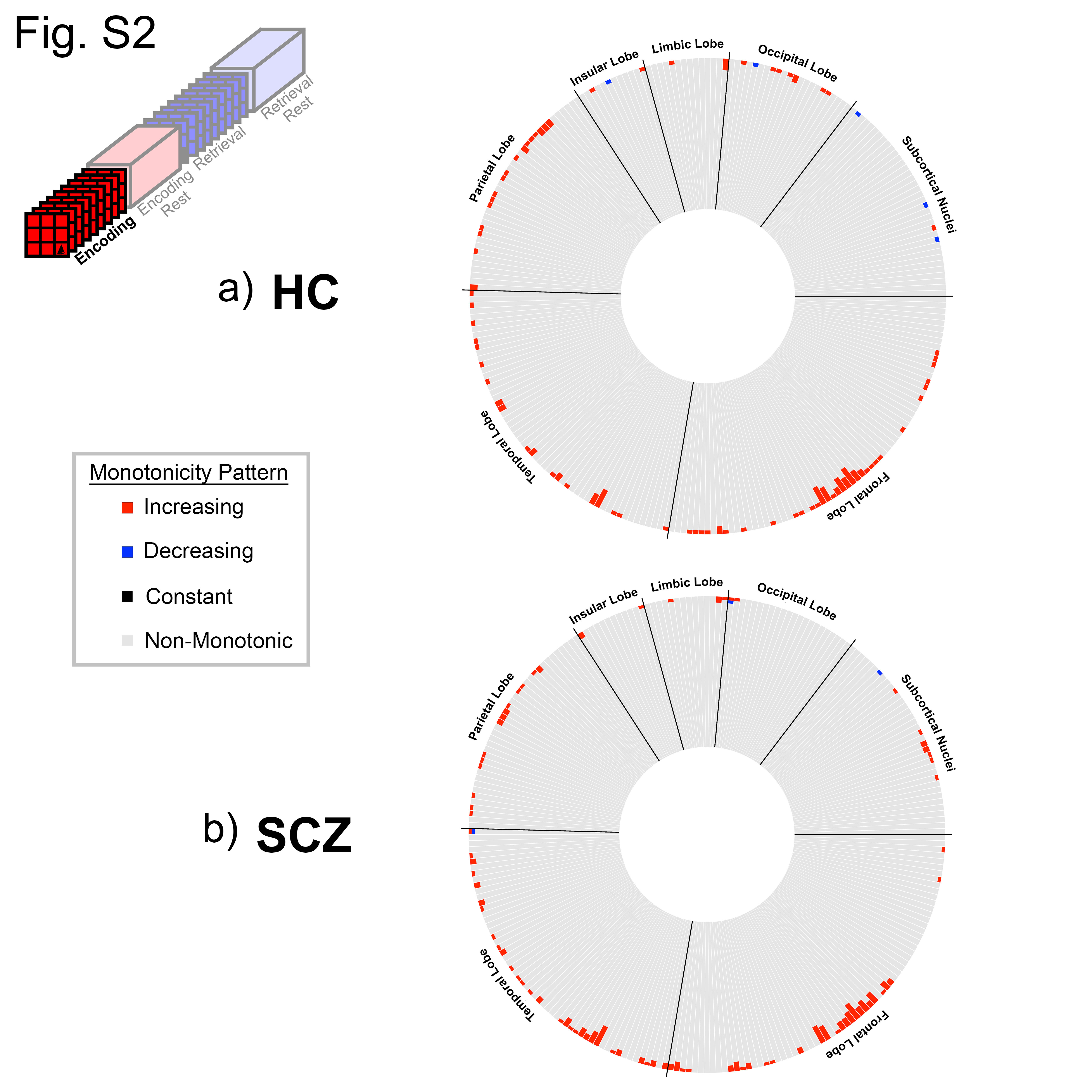

Supplement: SUPPLEMENTARY FIGURE 2 — Monotonicity of fMRI signal amplitudes during Encoding at the participant level for (a) HC and (b) SCZ. The figure complements Figure 3a and the arrangement is maintained. As was the case with the findings from BCRO, non-monotonicity was the norm in a majority of participants. This sparsity is also seen in Supplementary Figures 3–5. [file Image_2.jpeg]

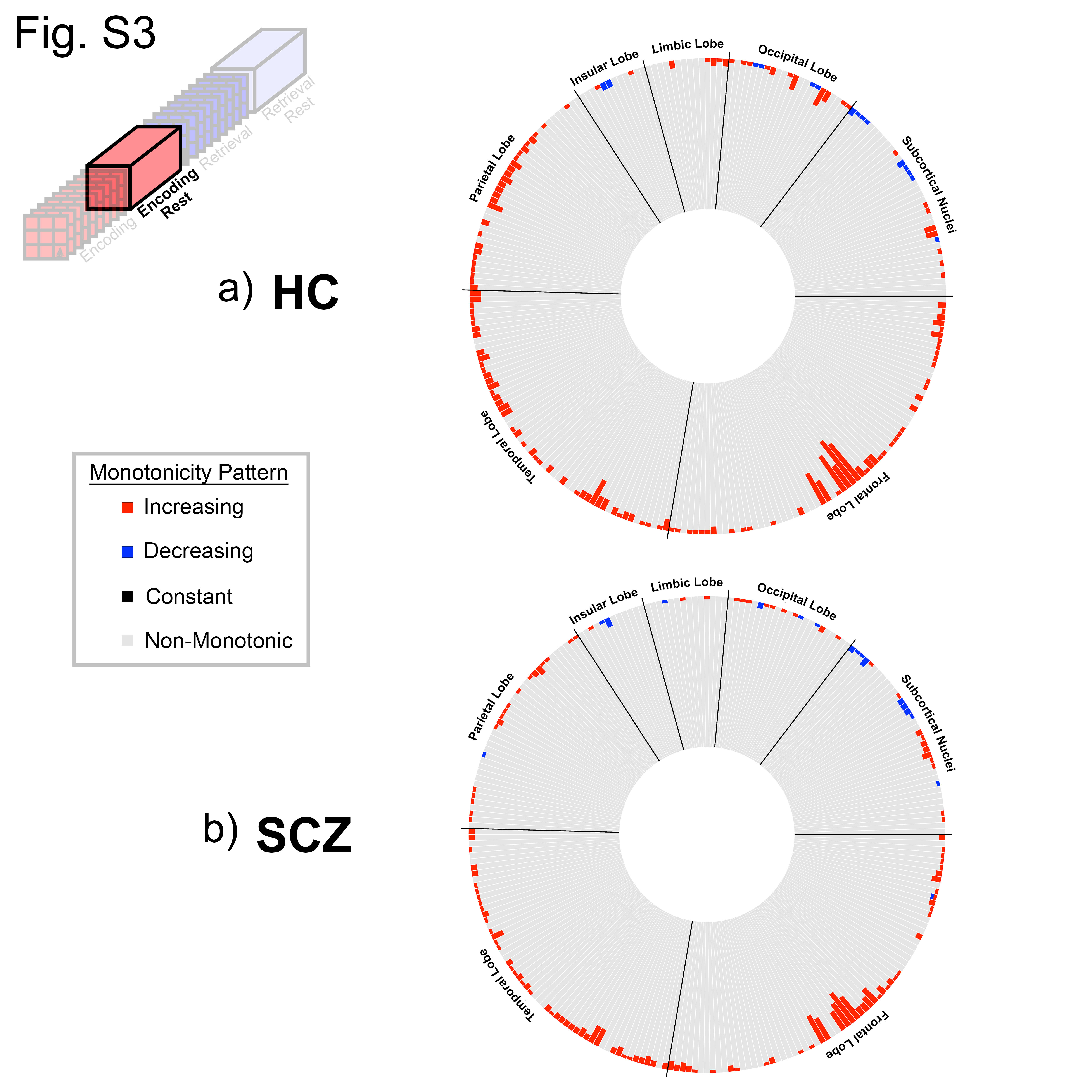

Supplement: SUPPLEMENTARY FIGURE 3 — Monotonicity of fMRI signal amplitudes during Post-Encoding Rest at the participant level for (a) HC and (b) SCZ. The figure complements Figure 4a. [file Image_3.jpeg]

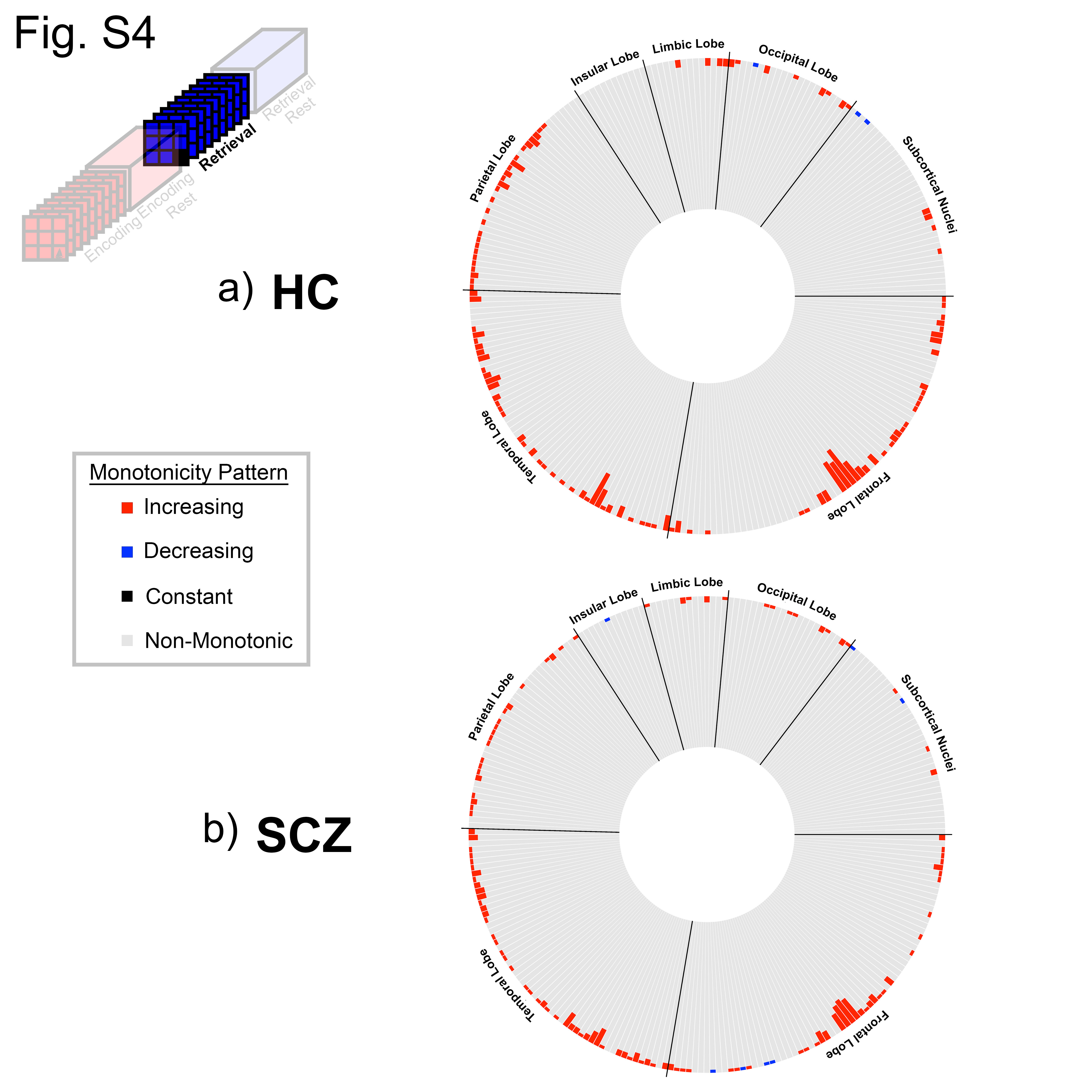

Supplement: SUPPLEMENTARY FIGURE 4 — Monotonicity of fMRI signal amplitudes during Retrieval at the participant level for (a) HC and (b) SCZ. The figure complements Figure 5a. [file Image_4.jpeg]

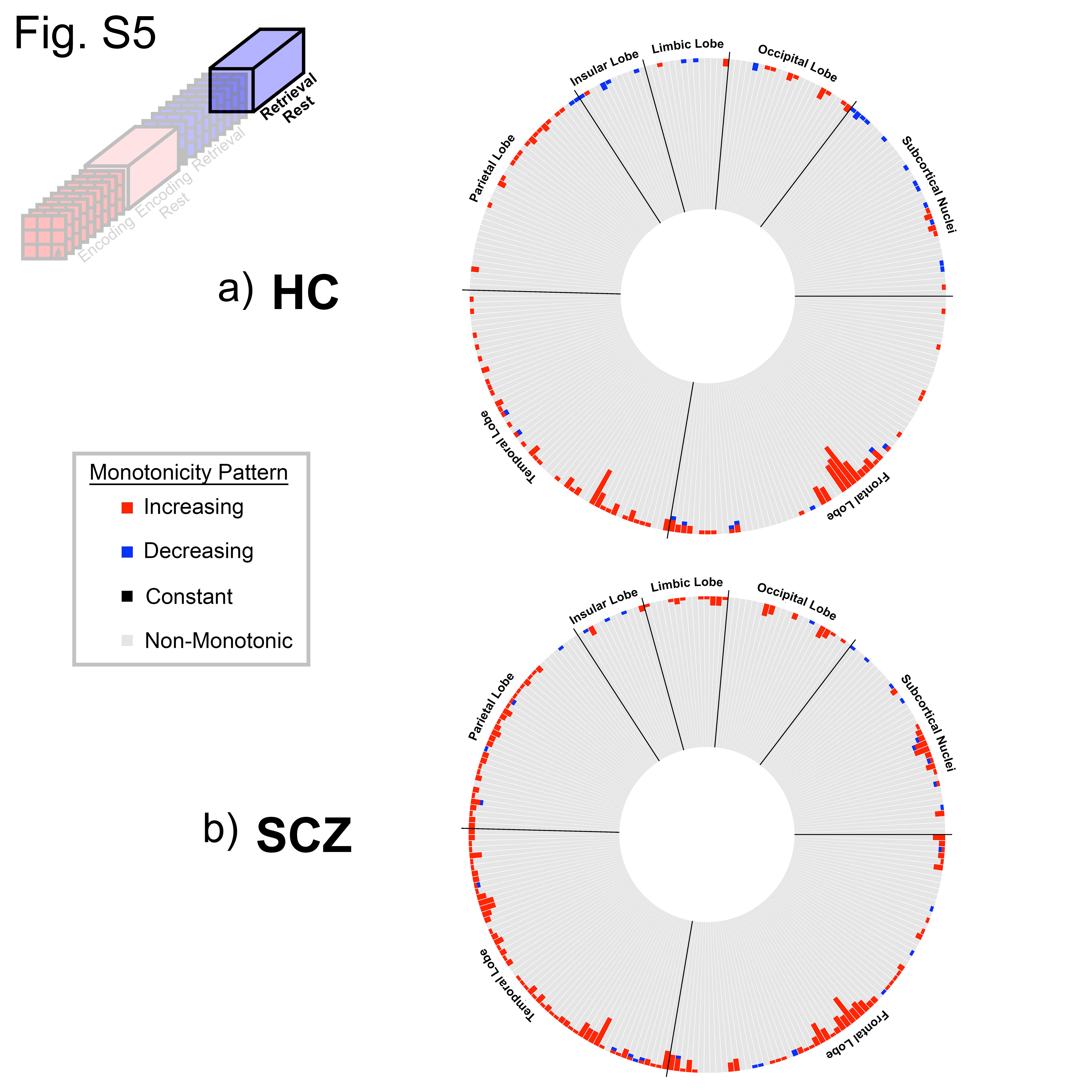

Supplement: SUPPLEMENTARY FIGURE 5 — Monotonicity of fMRI signal amplitudes during Post-Retrieval Rest at the participant level for (a) HC and (b) SCZ. The figure complements Figure 6a. [file Image_5.jpeg]

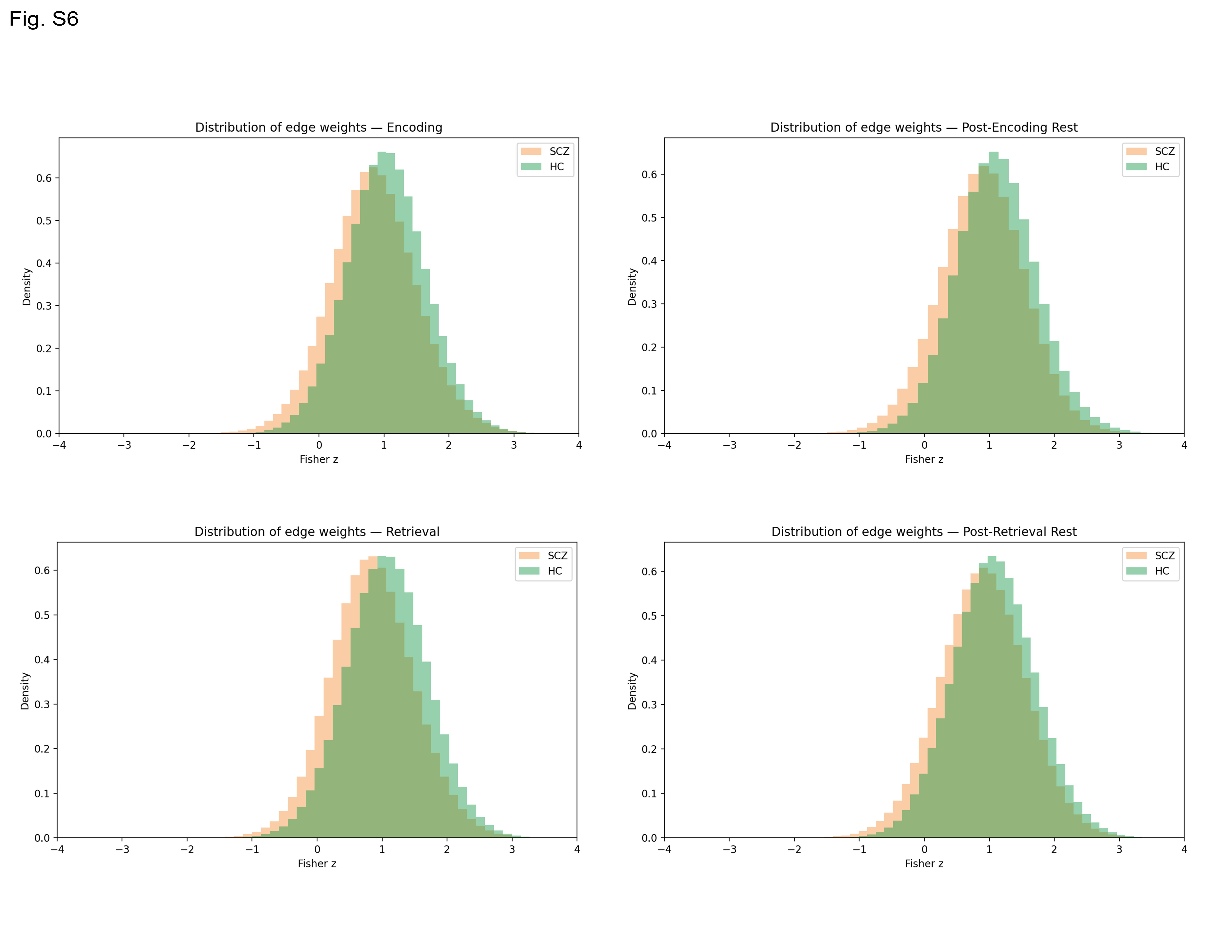

Supplement: SUPPLEMENTARY FIGURE 6 — Distributions of edge-weight (Fisher z transformed correlation coefficients derived from the uFC matrices) across the eight iterations for each task condition in HC and SCZ. [file Image_6.jpeg]
