# Supplementary material for: Monotonicity in graph theoretic summaries of fMRI data acquired during human learning
Source: Front Hum Neurosci. 2025 Sep 22;19:1595331. doi: 10.3389/fnhum.2025.1595331 (PMC12497747; doi:10.3389/fnhum.2025.1595331)
Supplement: Supplementary file 7 [file Data_Sheet_1.pdf]

Appendix

Defining expected frequencies of monotonic changes in BC_{RO}

We define monotonicity to be satisfied when across the eight time points (T_n, T_1 – T_8), the difference between at least six of the seven successive transitions has the same sign. We defined our expected frequencies under the assumption that the probability of an increase or decrease in BC_{RO} over successive T_n is 0.5. Thus, the probability that any region's BC_{RO} is monotonic by chance can be described as follows (Eq. 3).

$$p(region\ is\ monotonic) = p(6\ monotonic\ trans) + p(7\ monotonic\ trans) \quad \text{(A1)}$$

Using the probability mass function of a binomial distribution, we obtain (Eq. 4).

$$p(k) = \binom{n}{k}(p^k)(1-p)^{n-k} \quad \text{(A2)}$$

Where we define the probability of any region increasing or decreasing in BC_{RO} over each transition as .5 we obtain (Eq. 5).

$$= \binom{7}{6}(0.5^6)(1-0.5)^{7-6} + \binom{7}{7}(0.5^7)(1-0.5)^{7-7} \quad \text{(A3)}$$

$$= 0.0625$$

Therefore, by chance, we would expect 6.25% of our regions to exhibit weak monotonicity. Thus, in any participant, and in any condition, for our 246 region parcellation scheme, we would by chance expect to observe monotonicity in ~15 regions.
